# Importance of the Ion-Pair Lifetime in Polymer Electrolytes

**DOI:** 10.1021/acs.jpclett.1c02474

**Published:** 2021-08-27

**Authors:** Harish Gudla, Yunqi Shao, Supho Phunnarungsi, Daniel Brandell, Chao Zhang

**Affiliations:** Department of Chemistry-Ångström Laboratory, Uppsala University, Lägerhyddsvägen 1, Box 538, 75121 Uppsala, Sweden

## Abstract

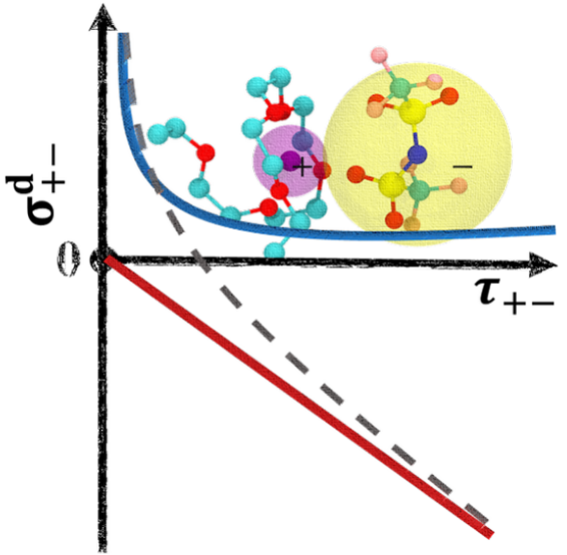

Ion pairing is commonly considered as a culprit for the reduced ionic conductivity in polymer electrolyte systems. However, this simple thermodynamic picture should not be taken literally, as ion pairing is a dynamical phenomenon. Here we construct model poly(ethylene oxide)–bis(trifluoromethane)sulfonimide lithium salt systems with different degrees of ion pairing by tuning the solvent polarity and examine the relation between the cation–anion distinct conductivity σ_+–_^d^ and the lifetime of ion pairs τ_+–_ using molecular dynamics simulations. It is found that there exist two distinct regimes where σ_+–_^d^ scales with 1/τ_+–_ and τ_+–_, respectively, and the latter is a signature of longer-lived ion pairs that contribute negatively to the total ionic conductivity. This suggests that ion pairs are kinetically different depending on the solvent polarity, which renders the ion-pair lifetime highly important when discussing its effect on ion transport in polymer electrolyte systems.

Ion pairing in electrolyte solutions^1−6^ results from a delicate balance between ion–solvent and ion–ion interactions. One common approach to define an ion pair is to use Bjerrum’s criterion,^7^ in which the distance *r*_+–_ is smaller than the effective range of −*q*_+_*q*_–_/2*ε k*_b_T (half of the Bjerrum length) with ε as the dielectric constant of the solution, *q*_+_ and *q*_–_ being the ionic charges, the Boltzmann constant *k*_b_, and the temperature *T*. Bjerrum’s criterion suggests that the solvent polarity plays a critical role in the formation of ion pairs, rendering a distinction between contact ion pairs (CIPs) and solvent-separated ion pairs (SSIPs).^1^ In addition, it implies that the formation of pairs of equal ionic species is unlikely to occur due to the electrostatic repulsion but that the possibility of forming triplets or larger aggregates, for example, an anion–cation–anion cluster, cannot be excluded.^8,9^

The idea that ion pairing affects the ionic conductivity was introduced early on by Arrhenius, who ascribed the decrease of the equivalent conductivity at a higher concentration to the formation of charge-neutral ion pairs.^10^ This idea has been put forward using the molar conductivity ratio Λ_EIS_/Λ_NMR_ measured by electrochemical impedance spectroscopy (EIS) and pulse-field gradient NMR to quantify the ionicity (the degree of dissociativity), particularly for ionic liquids^11,12^ and polymer electrolytes.^13^ Nevertheless, it has been realized that deviations of the ionic conductivity from the Nernst–Einstein relation cannot solely be attributed to the formation of ion pairs,^14−16^ where other factors such as the hydrodynamic interactions manifested via viscosity can play an important role.^17^

To describe the effect of ion pairing on the ionic conductivity, one needs an observable that can be accessed both theoretically and experimentally. The key quantity used here is the cation–anion distinct conductivity σ_+–_^d^ from liquid-state theory^18,19^
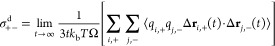
1where Ω is the volume of the system, and Δ**r**(*t*) is the displacement vector of each ion at time *t*. Note that σ_+–_^d^ is experimentally measurable^20,21^ and directly related to the Onsager transport coefficient Ω_+–_.^19,22^

Somewhat unexpectedly, σ_+–_^d^ is often found positive (instead of negative as in Arrhenius’ picture) in different types of electrolyte systems, spanning categories from aqueous electrolyte solutions to polymer ionic liquids.^15,17,23−27^ This suggests that the existence of ion pairs, as evinced by a number of spectroscopic experiments,^28−30^ does not necessarily imply a negative contribution to the measured ionic conductivity but can instead contribute to an increase in the transport of ions. Therefore, understanding the ion pairing effect on polymer electrolytes is crucial, as their application in energy storage systems is largely limited by a low ionic conductivity.^31−33^

The crucial point to this conundrum lies in the fact that Bjerrum’s convention is a thermodynamic criterion, while the ionic conductivity is a dynamical property. Therefore, the lifetime of charge-neutral ion pairs needs to be considered explicitly when discussing the contribution of ion pairing to the ionic conductivity, in addition to the distance criterion due to the thermodynamic stability. In other words, an ion pair should be “long-lived enough to be a recognizable kinetic entity”.^34^

Theoretically, the lifetime of ion pairs τ_+–_ can be extracted from the normalized time correlation function of the cation–anion pairs in molecular dynamics (MD) simulations^35^
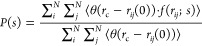
2where *f*(*r*_*ij*_;*s*) is a window function to detect whether a cation–anion pair lies within the cutoff *r*_c_ for a given period *s*.

The first approach is to use the product of the Heaviside functions θ(*x*) defined by a time series of pairwise distances *r*_*ij*_ between a cation–anion pair, as follows.^36^
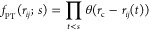
3However, the persistence time (PT) from this procedure clearly neglects recrossing events, for example, reactions passing over the transition state but returning to the reactant afterward, which has been discussed extensively for hydrogen-bond dynamics.^35,37^ Here, we used the stable states picture (SSP) of chemical reactions proposed by Laage and Hynes, which remedies this problem.^38^ Then *f*(*r*_*ij*_; *s*) in SSP is given as
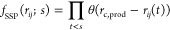
4where *r*_c,prod_ is the product SSP boundary, which corresponds to the cation–anion distance at the half height of the second peak in the radial distribution function (RDF). Then, *r*_c_ in eq 2 should be replaced by the reactant SSP boundary *r*_c,reac_, which is at the first maximum of the cation–anion RDF.

To investigate the relation between σ_+–_^d^ and τ_+–_ in polymer electrolyte systems, we constructed simulation boxes consisting of 200 poly(ethylene oxide) (PEO) polymer chains each with 25 ethylene oxide (EO) repeating units and 400 bis(trifluoromethane)sulfonimide lithium salt (LiTFSI) ([Li^+^]/[EO] concentration = 0.08). As indicated by Bjerrum’s criterion, the solvent polarity strongly modulates the ion pairing. This motivated us to apply the charge scaling method^39^ to PEO molecules to change the degree of ion pairing. General AMBER force field (GAFF)^40^ parameters were used for describing bonding and nonbonding interactions in PEO and LiTFSI, and all MD simulations were performed using GROMACS 2018.1.^41^ All systems were properly equilibrated to make sure that the simulation length is larger than the Rouse time of the polymer. Details for the system setup and MD simulations can be found in the Supporting Information Section A.1.

Before discussing our main result, it is necessary to check how structural and transport properties change when we tweak the handle of the solvent polarity. Here, the solvent polarity is described by the dielectric constant of the system ε_P_, which was computed for each polymer electrolyte system (see Section A.2 in the Supporting Information for details).

The RDFs of Li–N(TFSI) are plotted in Figure 1a, where peaks in the Li–N(TFSI) RDF increase significantly when ε_P_ becomes smaller. This is a sign of formation of ion pairs, which is also evinced in Figure 2. Accordingly, there is an optimal value in the total Green–Kubo conductivity σ_G–K_ when the solvent polarity is modulated as seen in Figure 1b. Both of these results support our previous observations of the effect of solvent polarity on the Li^+^ transportation in PEO-LiTFSI systems^42^ and agree with other recent studies of polymer electrolyte systems.^43,44^

**Figure 1:**
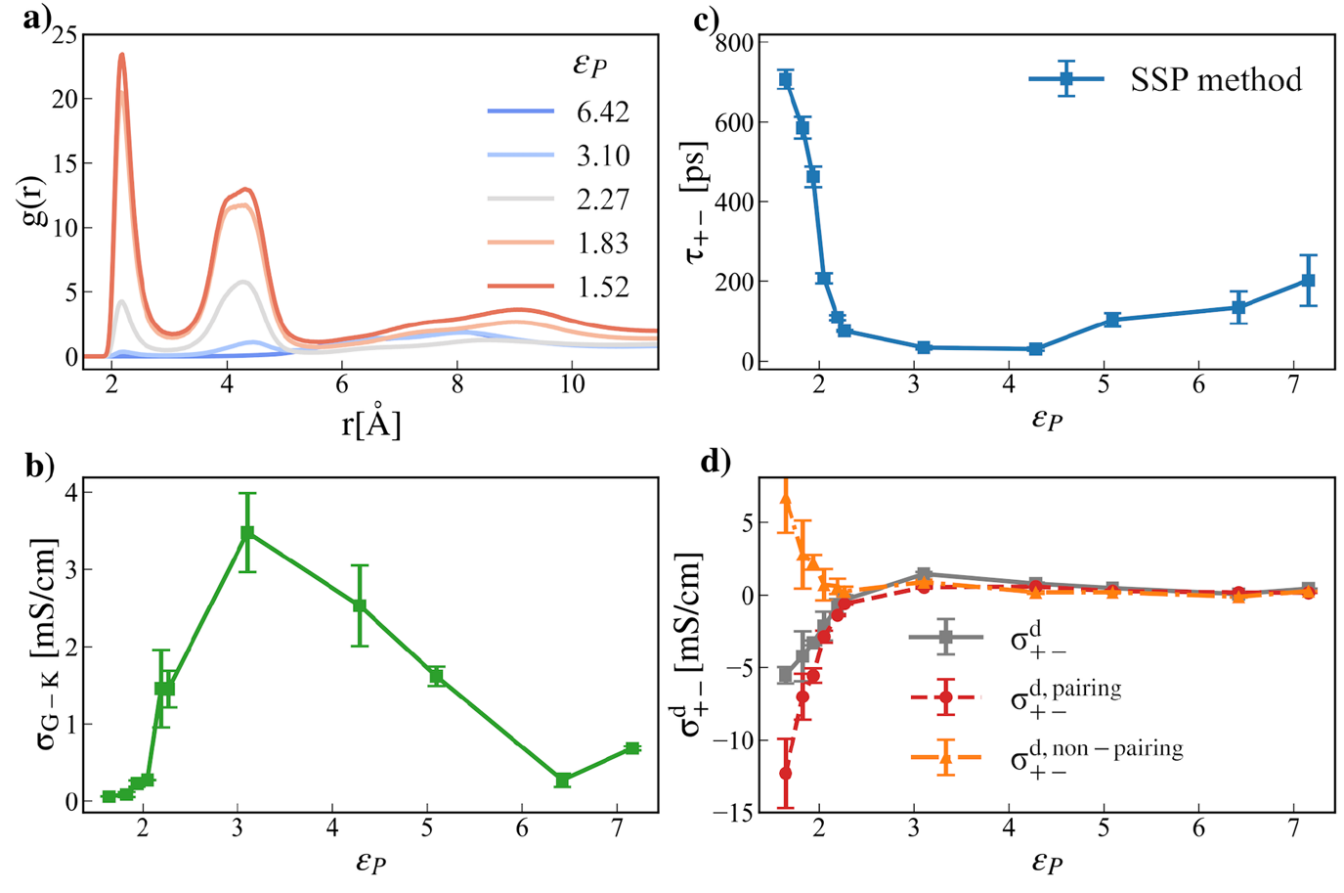
(a) The Li–N(TFSI) RDF at different solvent polarity strengths (as quantified by the dielectric constant ε_P_). (b) The total conductivity σ_G-K_ computed from the Green–Kubo relation as a function of ε_P_. (c) The lifetime of ion pairs τ_+–_ computed from the SSP method as a function of ε_P_, where *r*_c,reac_ = 2.1 Å and *r*_c,prod_ = 3.8–5.5 Å. (d) The cation–anion distinct conductivity σ_+–_^d^ (and its decomposition into pairing and non-pairing contributions) as a function of ε_P_.

**Figure 2:**
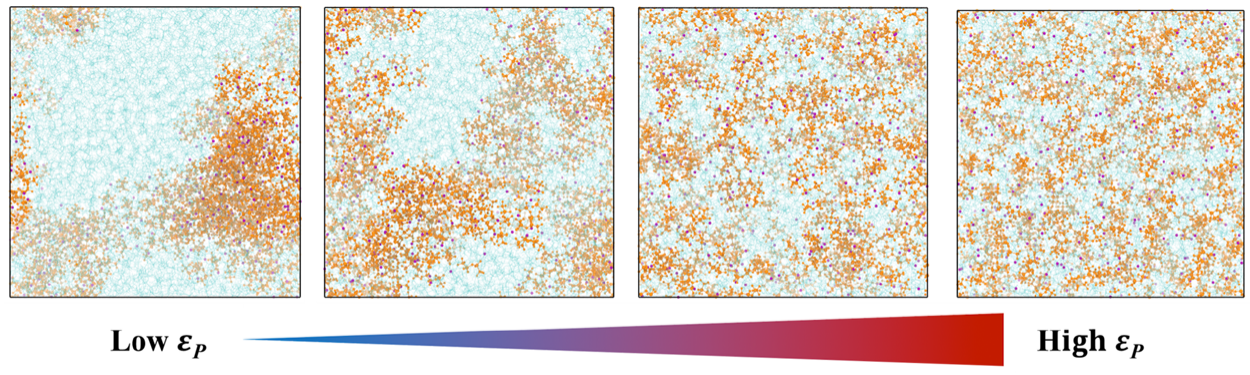
Modulation of ion pairing in PEO-LiTFS systems by solvent polarity ε_P_. Sky blue - PEO chains, Purple - Li ions, Orange - TFSI ions.

Figure 1c,d, however, demonstrates novel phenomena. The lifetime of ion pairs increases when ε_P_ is either high or low, and it reaches a minimum at the intermediate value of ε_P_ (see Section A.3 in the Supporting Information for further details of calculations of the lifetime of ion pairs and a comparison of outcomes from eq 3 and eq 4). Inspecting Figure 1b,c, one may attempt to relate the opposite trend shown in the total ionic conductivity σ_G–K_ to that of τ_+–_. However, the lifetime increases much more rapidly at a lower dielectric constant regime (ε_P_ < 2.3) compared to that at a higher dielectric constant regime (ε_P_ > 3). This suggests there are different types of ion pairs in polymer electrolyte systems under investigation here. Looking at the cation–anion distinct conductivity σ_+–_^d^, one can clearly see that it goes from positive to negative when ε_P_ becomes smaller. (Note that σ_+–_^d^ > 0 corresponds to anticorrelated cation–anion movements for the sign convention used in this work.) In particular, the rapid decrement in σ_+–_^d^ at lower ε_P_ seems in accord with the rapid increment in τ_+–_. These observations also point to the direction that these two key properties of ion pairs, namely, σ_+–_^d^ and τ_+–_, must be closely related.

This leads to our main result shown in Figure 3. What we find is that there exist two distinct regimes: σ_+–_^d^ scales with 1/τ_+–_ (for higher values of ε_P_), and σ_+–_^d^ scales with τ_+–_ (for lower values of ε_P_). Moreover, the transition between these two regimes shows a combined feature. Therefore, the general scaling relation we propose for polymer electrolyte systems is
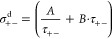
5where both *A* and *B* are system-dependent coefficients. Therefore, what matters to discussions of the ion-pairing effect on transport properties in polymer electrolytes is not whether ion pairs are present or not in the system but how long they live. By establishing the scaling relation for ion pairs from MD simulations, one could predict the lifetime of ion pairs using the measured value of σ_+–_^d^ in experiments.^27^

**Figure 3:**
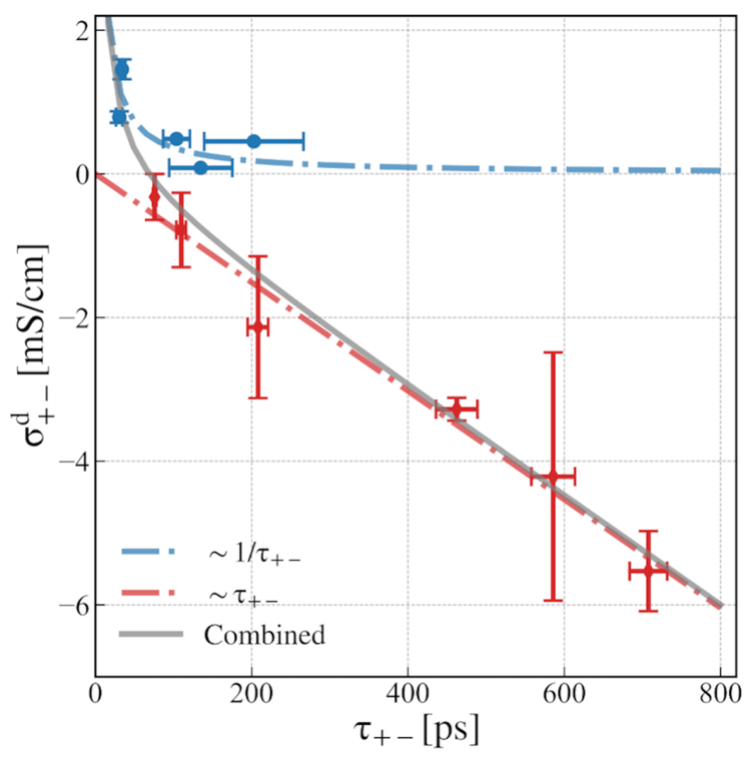
Scaling relation between the cation–anion distinct conductivity σ_+–_^d^ and the lifetime of ion pairs τ_+–_ computed from the SSP method for PEO-LiTFSI polymer electrolyte systems with different solvent polarity strengths.

Then, the immediate question that appears is, Why do shorter-lived ion pairs scale with 1/τ_+–_, while longer-lived counterparts scale with τ_+–_? The first scaling relation seems rather general, as already observed in ionic liquids, organic electrolytes, polymer ionic liquids, and salt-doped homopolymers.^23,26,45−47^ This is reminiscent of the Walden rule or the Stokes–Einstein relation. The second scaling relation we find in this work is a consequence of that τ_+–_ computed from the SSP method is equal to the inverse of the reactive flux rate constant 1/*k*_RF_^38^ for the ion-pair dissociation, and 1/*k*_RF_ is proportional to the concentration of ion pairs from the law of mass action. Since σ_+–_^d^ is also proportional to the number of ion pairs, this leads to the observation that σ_+–_^d^ scales linearly with τ_+–_.

It is worth mentioning that σ_+–_^d^ includes both contributions from the longer-lived ion pairs and the remainder. This suggests that one could further separate these two contributions for longer-lived pairs
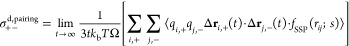
6where *f*_SSP_ is the same function given by eq 4. Then, the contribution from the remainder is simply σ_+–_^d, nonpairing^ = σ_+–_^d^ – σ_+–_^d, pairing^. Here, the parameter *s* is chosen to be 2 ns, as decided by the convergence of the conductivity calculation (see Section A.4 in the Supporting Information).

The result of this decomposition is shown in Figure 1d. The σ_+–_^d, pairing^ remains zero until a lower value of ε_P_. This agrees with the appearance of longer-lived ion pairs as seen in Figure 1c. More interestingly, in the presence of longer-lived ion pairs, the σ_+–_^d, pairing^ is negative, but the σ_+–_^d, nonpairing^ is positive instead. To understand why, we made a toy model of a NaCl solution where all Na–Cl are paired up with holonomic constraints. Details for the system setup and MD simulations can be found in Section B of the Supporting Information.

Mean square charge displacements (MSCD, i.e., quantities inside the square bracket in eq 1 and eq 6) of this toy model are shown in Figure 4, for the total ionic conductivity σ_G-K_, self-conductivities (σ_+_ + σ_–_), and the sum of cation–cation and anion–anion distinct conductivities (σ_++_^d^ + σ_––_^d^) as well as σ_+–_^d, pairing^ and σ_+–_^d, nonpairing^. Since all Na–Cl ion pairs are permanent by construction, the total ionic conductivity as the sum of all these individual contributions mentioned above must be zero (i.e., the slope of the MSCD “total” is zero), as evinced in Figure 4. Moreover, self-conductivities (σ_+_ + σ_–_) should be exactly the negative of the direct part of the cation–anion distinct conductivity σ_+–_^d, pairing^, as seen also in Figure 4. On the basis of these considerations, we know that the sum of σ_++_^d^, σ_––_^d^, and σ_+–_^d, nonpairing^ is zero as well. As shown in Figure 4, σ_++_^d^ and σ_––_^d^ are negative, while σ_+–_^d, nonpairing^ is positive in the toy model with permanent ion pairs. This provides a rationale to the opposite signs of σ_+–_^d, pairing^ and σ_+–_^d, nonpairing^ as seen in Figure 1d of PEO-LiTFSI systems. Nevertheless, one should be aware that the situation with ion aggregates will be different, as σ_++_^d^ and σ_––_^d^ could be positive instead.

**Figure 4:**
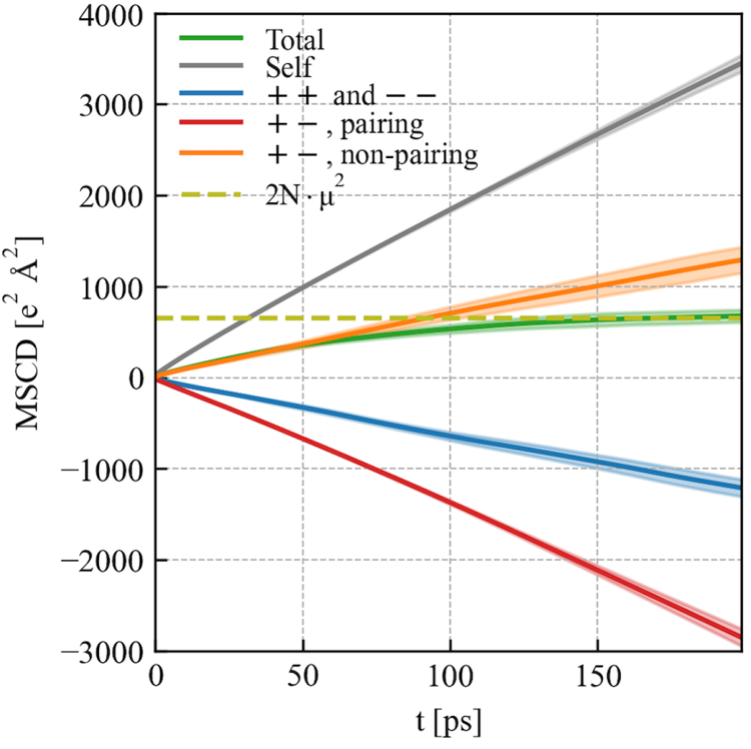
MSCD of a 5 mol NaCl solution with permanent ion pairs (in the same order as those that appeared in the key box): for the total ionic conductivity σ_G-K_, the self-conductivity (σ_+_ + σ_–_), the sum of cation–cation and anion–anion distinct conductivities (σ_++_^d^ + σ_––_^d^), the ion pairing part of the cation–anion distinct conductivity σ_+–_^d, pairing^, and the remainder part σ_+–_^d, nonpairing^. *N* is the number of ion pairs in the system, and μ is the dipole moment of each ion pair.

To sum up, following Bjerrum’s criterion, we have constructed PEO-LiTFSI systems in our MD simulations with different degrees of ion pairing by modulating the solvent polarity. What we found is that there exist two distinct regimes where the cation–anion distinct conductivity σ_+–_^d^ scales with 1/τ_+–_ and with τ_+–_, respectively. The linear scaling of σ_+–_^d^ with respect to the lifetime of the ion pairs τ_+–_ is a signature of longer-lived ion pairs that reduce the total ionic conductivity. By establishing this scaling relation, one could infer the lifetime of ion pairs from the experimentally measured cation–anion distinct conductivity. This further suggests that what matters to discussions of the ion-pairing effect on transport properties in polymer electrolytes is not the presence of ion pairs but the corresponding lifetime.

In the scaling relation we found in our MD simulations (eq 5), the coefficient *A* is positive, which suggests anticorrelated movements of cation–anions and shorter-lived ion pairs. This hints that ion aggregates analyzed in previous MD studies of the PEO-LiTFSI system^48^ would not populate in this scenario, in line with conclusions drawn from other experimental works for polymer electrolytes.^49,50^ Nevertheless, it is worth noting that early experiments on aqueous ionic solutions show that σ_+–_^d^ can flip the sign from negative (correlated) to positive (anticorrelated) when the salt concentration is increased,^20^ which is intriguing. This clearly indicates that the cation–anion distinct conductivity σ_+–_^d^ is a sensitive probe to the convoluted ion–ion correlations, which calls for further investigations from both experiments and simulations to understand its nature and its relationship with other static and dynamical properties in electrolyte systems.

## Supporting Information Available

Descriptions of the setup and MD simulations of PEO-LiTFSI systems and NaCl solution with permanent ion pairs (PDF)
