# Supplementary material for: Importance of the Ion-Pair Lifetime in Polymer Electrolytes
Source: J Phys Chem Lett. 2021 Aug 27;12(35):8460–4. doi: 10.1021/acs.jpclett.1c02474 (PMC8436209; doi:10.1021/acs.jpclett.1c02474)
Supplement: Supplementary file 1 — jz1c02474_si_001.pdf [file jz1c02474_si_001.pdf]

Supporting Information

Importance of the Ion-Pair Lifetime in Polymer Electrolytes

Harish Gudla[†], Yunqi Shao[†], Supho Phunnarungsi[†], Daniel Brandell[†] and Chao Zhang[†*]

[†]*Department of Chemistry - Ångström Laboratory, Uppsala University, Lägerhyddsvägen 1, BOX 538, 75121, Uppsala, Sweden*

chao.zhang@kemi.uu.se

A Description of the setup and MD simulations of PEO-LiTFSI systems

A.1 Force field and simulation details

General AMBER force field (GAFF)[1] parameters were used for describing bonding and non-bonding interactions in PEO and LiTFSI and all simulations were performed using GROMACS 2018.1.[2]

PEO		LiTFSI	
Atom type	Charge (e)	Atome type	Charge (e)
C	0.13	C	0.41
O_{chain}	-0.43	S	1.11, 1.26
H_{chain}	0.04	F	-0.20
O_{end}	-0.61	N	-0.79
H_{end}	0.41	O	0.46,0.49
		Li	0.75

Table S1: Intial partial charges of PEO from GAFF and scaled charges of LiTFSI from GAFF.

MD simulation boxes comprising neat and LiTFSI-doped PEO systems were constructed, comprising 200 hydroxyl-terminated chains, each with 25 monomer units (1.11 kg/mol) and 400 Li and 400 TFSI ions, corresponding to a [Li+]/[EO] concentration ratio of 0.08. All systems were equilibrated using a Bussi-Donadio-Parrinello[3] thermostat and a Parrinello-Rahman barostat[4] at 400 K and 1 bar for 5 and 10 ns, respectively with a time step of 1 fs and snapshots saved every 5 ps. Then, NPT (constant number of particles, constant pressure, and constant temperature) production runs were carried out for additional 400 ns at a desired temperatures. The specific temperatures were chosen to separate the effect of glass transition temperature and solvent polarity on the polymer and ion dynamics.[5] The dielectric constant of the system was estimated from the fluctuations in the total dipole moment of the PEO was used as a measure of the solvent polarity. The solvent polarity of the system can be modulated by scaling the partial atomic charges on polymer. The scaling factor and dielectric constant of different systems used in this work is reported in Table S2.

A.2 Calculation of the dielectric constant of polymer electrolyte systems

The dielectric constant of the polymer electrolyte ε_P reflects the strength of solvent polarity. The dielectric constant of PEO-LiTFSI system with periodic boundary conditions and Ewald summation can be computed from the fluctuations in the total dipole moment $\mathbf{M}$ of the PEO via the equation for a non-polarizable model[6]:

$$\varepsilon_p = 1 + \frac{4\pi}{3\varepsilon_0 \Omega k_B T}(\langle \mathbf{M}^2 \rangle - \langle \mathbf{M} \rangle^2) \tag{S1}$$

where ε_0 is vacuum permittivity, k_B is Boltzmann's constant, T is temperature, Ω is the average volume of the simulation box and $\langle\ \rangle$ indicates ensemble averages.

Scaling factor	T (K)	ε_P	box size (nm^3)
1.55	450	7.16 (0.22)	438.33
1.50	450	6.42 (0.11)	440.58
1.34	440	5.09 (0.03)	442.77
1.20	440	4.29 (0.02)	447.30
1.00	430	3.10 (0.02)	450.29
0.75	420	2.27 (0.05)	453.97
0.70	420	2.19 (0.00)	455.04
0.65	420	2.05 (0.02)	456.78
0.60	410	1.94 (0.00)	454.98
0.56	410	1.83 (0.03)	456.76
0.50	410	1.65 (0.00)	458.74

Table S2: Details of the different simulated systems.

A.3 Calculation of lifetime of ion-pairs and comparison of methods

The lifetime of ion-pairs can be extracted from the normalized time correlation function representing the life expectancies of the pairs, i.e. the first passage time probability function[7]:

$$P(s) = \langle \theta(r_c - r_{ij}(0)) \cdot f(r_{ij}; s) \rangle / \langle \theta(r_c - r_{ij}(0)) \rangle \tag{S2}$$

where $f(r_{ij}; s)$ can be calculated from Eq. 3 for persistence lifetime (τ_{+-}^{PT}) or from Eq. 4 for stable state picture lifetime (τ_{+-}^{SSP}) (See the Main Text for Eq.3 and Eq.4). The lifetime can then be obtained by fitting $P(s)$ to a biexponentially decaying function which converges to a constant.

$$P(s) = (1 - A_1 - A2) + A_1 \exp{(-\frac{s}{\tau_1})} + A_2 \exp{(-\frac{s}{\tau_2})} \tag{S3}$$

$$\tau_{+-} = \frac{A_1\tau_1 + A_2\tau_2}{A_1 + A_2} \tag{S4}$$

The normalized time correlation function ($P(s)$) of Li and N(TFSI) were calculated for a correlation time of 5 ns for every 20 ns segment of the trajectory (See Fig. S1(a)). The ion-pair lifetime values from both the methods τ_{+-}^{PT} and τ_{+-}^{SSP} were plotted in Fig. S1(b), we can observe that the persistence lifetimes were much lower than the SSP lifetime because of missing out the recrossing events.

A.4 Convergence of the conductivity calculations

The Green-Kubo (G-K) formula that was used to calculate the total ionic conductivity is:

$$\sigma_{\text{G-K}} = \lim_{t\to\infty} \frac{1}{6tk_bT\Omega} \left[\sum_i^N \sum_j^N \langle q_i q_j \Delta\mathbf{r}_i(t) \cdot \Delta\mathbf{r}_j(t) \rangle \right] \tag{S5}$$

where Ω is the volume of the system and $\Delta\mathbf{r}(t)$ is the displacement vector of each ion at time t. The cumulative averages of instantaneous ionic conductivity values of $\sigma_{\text{G-K}}$ and σ_{+-}^d were used to calculate the reported values and were plotted for $\varepsilon_P = 2.19$ in Fig. S2(a). For all the systems, these values converges after 100 ns. Therefore, the final values were calculated

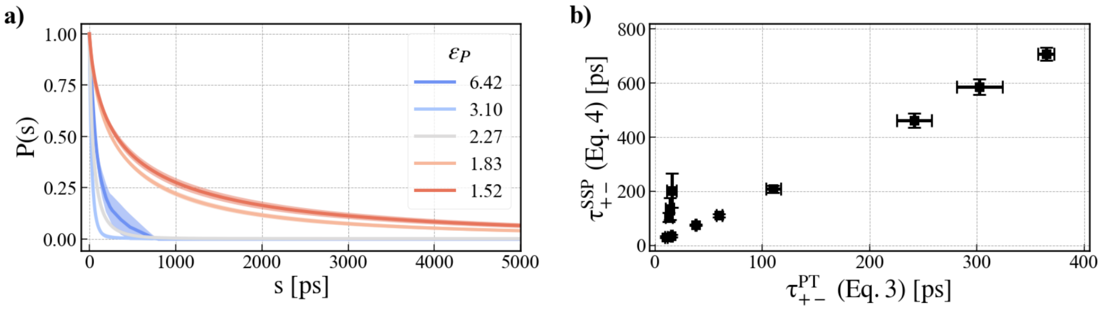

Figure S1: a) The normalized time correlation function ($P(s)$) of Li and N(TFSI) for sytems with differnt scaling factors or solvent polarity (ε_P). b) The pesistance lifetime (τ_{+-}^{PT}) vs stable state picture lifetime (τ_{+-}^{SSP}) at different solvent polarities.

by averaging over 120 ns to 140 ns. The convergence of $\sigma_{+-}^{\mathrm{d,pairing}}$ with r_c of 3.1 Å was also checked and plotted in Fig. S2(b) for PEO-LiTFSI system with different solvent polarities. The final values of $\sigma_{+-}^{\mathrm{d,pairing}}$ were calculated by averaging over 1.4 ns to 1.8 ns.

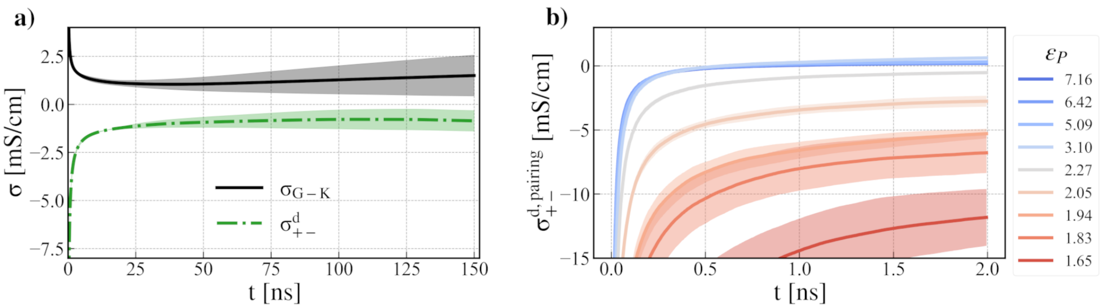

Figure S2: a) The instantaneous ionic conductivity values of $\sigma_{\text{G-K}}$ and σ_{+-}^{d} for the PEO-LiTFSI system with $\varepsilon_P = 2.19$. b) The instantaneous $\sigma_{+-}^{\mathrm{d,pairing}}$ for the PEO-LiTFSI sytem at different solvent polarities where ion-pairs are defined with a cutt-off distance (r_c) of 3.1 Å and time period (s) of 2 ns.

B Description of the setup and MD simulations of NaCl solution with permanent ion-pairs

Systems of sodium chloride electrolyte solution where the water molecules were described with the simple point charge/extended (SPC/E) model[8] and Na/Cl ions are described with parameters proposed by Joung and Cheatham[9]. To introduce permanent ion-pairs to the system, holonomic constraints through the SHAKE algorithm [10] were added between selected pair of ions, the bond length was set to be 2.85 Å. according to the position of the first peak in the Na-Cl RDF in the unconstrained system. The size, density and number of molecules used in the simulation are listed in Table S3.

The molecular dynamics simulations were performed with the LAMMPS code. The long-range electrostatics were computed using the particle-particle particle-mesh (PPPM) solver[11]. Short-range cutoffs for the van der Waals and Coulomb interactions in direct space were 9.8 Å. For each system, NVT (constant number of particles, constant volume, and constant temper-

m [mol kg^{-1}]	ρ [g cm^{-3}]	N_{NaCl}	$N_{\mathrm{H_2O}}$	L [Å]
5.14	1.17	40	432	24.29

Table S3: Size and composition of the MD simulations of NaCl solution in this work: molality m, density ρ, number of NaCl and H_2O molecules N_{NaCl} and $N_{\mathrm{H_2O}}$ and length of the cubic simulation box L.

ature) simulations ran for 20 ns with a timestep of 2 fs and trajectories were collected every 0.5 ps. The Bussi-Donadio-Parrinello thermostat[3] was used to maintain the given temperature of 293 K.

References

(1) Wang, J.; Wolf, R. M.; Caldwell, J. W.; Kollman, P. A.; Case, D. A. Development and testing of a general amber force field. *J. Comput. Chem.* **2004**, *25*, 1157–1174.

(2) Abraham, M. J.; Murtola, T.; Schulz, R.; Páll, S.; Smith, J. C.; Hess, B.; Lindah, E. GROMACS: High performance molecular simulations through multi-level parallelism from laptops to supercomputers. *SoftwareX* **2015**, *1-2*, 19–25.

(3) Bussi, G.; Donadio, D.; Parrinello, M. Canonical Sampling Through Velocity Rescaling. *J. Chem. Phys.* **2007**, *126*, 014101.

(4) Parrinello, M.; Rahman, A. Polymorphic transitions in single crystals: A new molecular dynamics method. *J. Appl. Phys.* **1981**, *52*, 7182–7190.

(5) Gudla, H.; Zhang, C.; Brandell, D. Effects of solvent polarity on Li-ion diffusion in polymer electrolytes: An all-atom molecular dynamics study with charge scaling. *J. Phys. Chem. B* **2020**, *124*, 8124–8131.

(6) Neumann, M. Dipole moment fluctuation formulas in computer simulations of polar systems. *Mol. Phys.* **1983**, *50*, 841–858.

(7) Luzar, A. Resolving the hydrogen bond dynamics conundrum. *J. Chem. Phys.* **2000**, *113*, 10663–10675.

(8) Berendsen, H. J. C.; Grigera, J. R.; Straatsma, T. P. The missing term in effective pair potentials. *J. Phys. Chem.* **1987**, *91*, 6269–6271.

(9) Joung, I. S.; Cheatham, T. E. Determination of alkali and halide monovalent ion parameters for use in explicitly solvated biomolecular simulations. *J. Phys. Chem. B* **2008**, *112*, 9020–9041.

(10) Ryckaert, J. P.; Ciccotti, G.; Berendsen, H. J. C. Numerical integration of the cartesian equations of motion of a system with constraints: molecular dynamics of n-alkanes. *J. Comput. Phys.* **1977**, *23*, 327–341.

(11) Hockney, R. W.; Eastwood, J. W., *Computer Simulation Using Particles*; CRC Press: New York, 1988.
